# Efficacy and Safety of Altibrain^®^ as an Adjunctive Therapy for Autism Spectrum Disorder: An Open Label Trial Targeting Core Symptoms

**DOI:** 10.2174/0113816128335544241210144541

**Published:** 2025-01-24

**Authors:** Sidharth Mehan, Aakash Kumar, Prashant R. Utage, Anaita Hegde, Neeta Naik, Santosh Kondekar, Nandan Yardi, Neelu Desai, Debasis Panigrahi, Arijit Chattopadhyay, Sasmita Devi Agarwal, Vineet Bhushan Gupta, Ankita Tiwari, Sai Chandar Reddy, Sandeep Saraf, Diptanshu Das, Mayank Detroja, Charu Paliwal

**Affiliations:** 1 Department of Pharmacology, Division of Neuroscience, ISF College of Pharmacy, Moga, Punjab, India (Affiliated to IK Gujral Punjab Technical University, Jalandhar, Punjab, 144603, India);; 2 Pediatric Neurology, Utage Child Development Centre, Hyderabad, India;; 3 Pediatric Neurology, SRCC Hospital, Mumbai, India;; 4 Pediatric Neurology, EN1 Neuroservices Pvt. Ltd, Mumbai, India;; 5 Pediatric Neurology, Aakar Clinic, Mumbai, India;; 6 Pediatric Neurology, Yardi Hospital, Pune, India;; 7 Pediatric Neurology, Hinduja Hospital, Mumbai, India;; 8 Pediatric Neurology, Ankur Hospital, Bhubaneshwar, India;; 9 Developmental Pediatrician, Apollo Multispecialty Hospitals, Kolkata, India;; 10 Pediatric Neurology, Hi-Tech Medical College & Hospital - Bhubaneswar, India;; 11 Pediatric Neurologist, Apollo Hospitals, Delhi, India;; 12 Pediatric Neurology, Lotus Hospital, Indore, India;; 13 Pediatric Neurology, Dr. Sai Chandar Neurocare, Warangal, India;; 14 Pediatric Neurology, Star Kids Hospital, Aurangabad, India;; 15 Pediatric Neurology, Institute of Neurodevelopment, Kolkata, India;; 16 Pediatric Neurology, Sahaj Child Neurology & Epilepsy Centre, Surat, India;; 17 Director Operations and Medical Writing, RYT Lifesciences Pvt Ltd, Ahmedabad, Gujrat, India

**Keywords:** Autism spectrum disorder (ASD), Altibrain**^®^**, social communication, repetitive behaviors, sulforaphane, stereotypical behavior

## Abstract

**Objective:**

This study aimed to evaluate the effectiveness and safety of Altibrain**^®^** in combination with standard Autism Spectrum Disorder (ASD) treatment compared to standard ASD treatment alone in individuals diagnosed with ASD.

**Methods:**

A randomized, open-label trial was conducted involving 120 participants aged 3 to 17 years, randomly assigned to either the Standard ASD Treatment group or the Altibrain**^®^** + Standard ASD Treatment group. Sixty patients were randomly allocated to each Standard ASD Treatment group or the Altibrain**^®^** + Standard ASD Treatment group. Participant allocation was done by computer-generated randomization. Participants had confirmed ASD diagnoses based on DSM-V or ICD-11 criteria and demonstrated moderate to severe core ASD symptoms. Informed consent was obtained from caregivers. A total number of 120 subjects were included, consisting of 71 male and 49 female patients. Participants received either standard ASD treatment alone or Altibrain**^®^** in addition to standard ASD treatment orally once daily for 24 weeks. A total of 7 study visits/24 weeks to analyze the intervention efficacy of the Standard ASD Treatment group or the Altibrain**^®^** + Standard ASD Treatment group. Primary outcomes included changes in core ASD symptoms measured by the Autism Diagnostic Observation Schedule (ADOS) and safety assessments. Secondary outcomes included alterations in social communication skills, reduction in repetitive behaviors, overall functional improvements, safety and tolerability of Altibrain**^®^**.

**Results:**

Altibrain**^®^** significantly improved qualitative deficits in social interaction and repetitive behaviors compared to standard ASD treatment alone (*p* < 0.0001). The Altibrain**^®^** + Standard ASD Treatment group demonstrated significant improvements in social functioning, social awareness, cognition, communication, and motivation compared to the Standard ASD Treatment group (*p* < 0.0001). Additionally, Altibrain**^®^** showed superior efficacy in reducing hyperactivity/noncompliance, inappropriate speech, irritability, lethargy/ social withdrawal, stereotypic behavior, and aberrant behavior compared to standard treatment alone (*p* < 0.0001). Additionally, Altibrain**^®^** exhibited a favorable safety profile as per the 4-week post-treatment safety follow-up.

**Conclusion:**

Overall, Altibrain**^®^** holds promise as a valuable therapeutic option for individuals with ASD and their families. Limitations of the study include neuroimaging and biomarkers analysis and larger cohort studies.

## 1 INTRODUCTION

Autism Spectrum Disorder (ASD) is a neurodevelopmental disorder characterized by a wide range of symptoms, including difficulties in social interaction, verbal and nonverbal communication, and repetitive behaviors. It presents as a spectrum, with each individual displaying a unique combination and severity of traits [1]. ASD exhibits intricate genetic and epigenetic characteristics contributing to its phenotypic manifestations, with no single genetic marker available for definitive diagnosis [2-4]. Consequently, ASD diagnosis relies on phenotypic assessment, as there are currently no validated laboratory tests. Evidence-based treatment options for ASD remain limited, and no FDA-approved pharmacological therapies effectively target either the core symptoms or the underlying pathophysiological processes associated with ASD [5, 6]. Emerging research has explored various pathways and mechanisms to identify potential interventions for improving these core symptoms [7]. Previous studies have demonstrated that the downregulation of Nrf2, a key transcription factor in cellular defense mechanisms, plays a significant role in ASD. Nrf2 levels and Nrf2/Keap1 activity are dysregulated and reduced to half in autistic children, leading to increased oxidative stress and decreased antioxidant capacity [8, 9]. Another study revealed overexpression of NF-κB in ASD children. Their Brain tissue samples expressed neuroglia activation and neuroinflammation throughout the brain's white matter, including the cerebellum, mid-frontal area, and cingulate gyrus. Spinal tap indicated a significant increase, 200 times higher inflammatory cytokine levels [10]. Recent evidence suggests mitochondrial dysfunction and altered energy metabolism contribute to the social and cognitive deficits observed in autism patients [11]. Clinicians have also observed that children with ASD usually behave normally during febrile episodes, indicating a potential behavior change due to the upregulation of heat shock proteins involved in folding and unfolding of proteins and translocating protein complexes. Furthermore, dysregulation of neuronal autophagy contributes to the accumulation of abnormal proteins and damaged cellular components, potentially impacting neuronal function and the development of ASD [12]. Recent research has highlighted the role of hyperactivation of mTORC in ASD pathophysiology, leading to translation dysregulation, decreased neuronal autophagy, neuroinflammation, apoptosis, and increased oxidative stress [13]. However, several physiological pathways linked to ASD may be modulated by the small phytochemical sulforaphane, which serves as a potential molecule supported by extensive *in vitro*, preclinical, and clinical evidence. These pathways include (a) redox metabolism and oxidative stress, (b) mitochondrial dysfunction, (c) immune dysregulation and neuroinflammation, (d) febrile illness and the heat shock response, and (e) synaptic dysfunction [14, 15].

The currently available treatments primarily aim to control symptoms rather than directly dealing with the fundamental causes of the illness. This emphasizes the urgent need for additional research to discover biomarkers and comprehensively understand the complex pathophysiological pathways contributing to ASD. It is essential to consider and deal with these aspects in order to achieve effective treatment. Sulforaphane, a chemical that has been extensively studied, has shown great potential in addressing essential mechanism of the pathophysiology of ASD. Glucoraphanin, the inactive precursor, is obtained from cruciferous vegetables such as cabbage and broccoli. It increases the expression of Nrf 2 genes, blocks NF-κB and proinflammatory cytokines, triggers neuronal autophagy, and reduces mitochondrial dysfunction. Prior experiments, which encompassed a randomized parallel double-blind controlled trial with 15-week open label therapy involving young children and a study involving young adults with ASD, exhibited notable improvements in behavior [16]. In addition, the combination of sulforaphane with antipsychotic medications like risperidone demonstrated further advantages in decreasing irritability, suggesting the potential of sulforaphane as an adjunctive therapy [17].

Nevertheless, the procedure of extracting sulforaphane from broccoli sprouts lacks standardization, posing difficulties in achieving a consistent and commercially viable form. In order to overcome this constraint, scientists developed a durable and patented variant of sulforaphane called Altibrain^®^. Moreover, a clinical trial was undertaken to assess the effectiveness and safety of a standardized synthetic sulforaphane (Altibrain^®^) formulation in the treatment of people with ASD, particularly in the Indian community.

## 2 MATERIALS AND METHODS

The Ethics Committee, Shree Hospital, Pune, approved the study on 15 Nov 2022 before performing any study activities. Before initiating the study, the investigators established a collaborative partnership with a local, non-public school specializing in educating children and young adults with autism and related neurodevelopmental disorders, encompassing ages 3 to 17. This innovative academic-school-parent partnership aimed to enhance overall care and communication among caregivers, clinical providers, and teachers. All children/families enrolled in the school (n = 120 at study initiation) were enrolled in the study through various channels, including email, informational letters, and informational sessions.

## 3 PARTICIPATION SELECTION CRITERIA

Male and female participants aged 3 to 17 years with a confirmed diagnosis of ASD based on DSM-V [18-20] or ICD-11 criteria. Moderate to severe core symptoms of ASD as determined by standardized assessments *e.g*., ADOS and on a stable medication regimen for at least four weeks prior to enrolment. Informed consent was provided as per the study requirements. Study Exclusion was defined as any significant medical or psychiatric condition that could interfere with study participation or safety. Any Known allergies or hypersensitivity to any components of Altibrain**^®^**. Current use of investigational drugs or participation in other clinical trials. Seizure disorder that is not well controlled. History of substance abuse or dependence within the past 12 months. Inability to comply with study procedures or follow-up visits.

## 4 STUDY PROCEDURE

Informed consent was obtained from the parents/caregivers of all study participants to ensure their understanding and commitment to the study requirements. The study was randomized and open-label. All clinicians, parents, and teachers were aware of the initiation and duration of treatment. The intervention was 24 weeks in addressing core symptoms of ASD. A total of 120 participants were recruited, with 60 participants randomly assigned to the Standard ASD Treatment group and 60 individuals to the Altibrain**^®^** + Standard ASD Treatment group. Randomization was achieved through computer-generated random numbers, ensuring an equal allocation ratio. The study participants received Standard ASD Treatment or Altibrain**^®^** + Standard ASD Treatment orally once daily for the entire 24-week treatment period as per the randomization allocation. The following dosage of sulforaphane was incorporated, *i.e*. for children weighing below 50 kg, dose 30 mg and for children weighing above 50 kg, dose was 60 mg.

Standard ASD treatment encompasses behavioral interventions (*e.g*., ABA) Aberrant behavior analysis, specialized education, and medication to address core symptoms, improve social skills, and manage co-occurring conditions like anxiety or aggression. Participants attended a total of 07 study visits described in Table **1**. (Visit 01: Screening visit (-21 Days), Visit 02: Day 01, Visit 03: 29 Days/Week 04, Visit 04: Day 85/Week 12, Visit 05: Day 127/Week 18, Visit 06: Day 169/Week 24/End of Treatment and Visit 07: 28 Days of EOT/Safety follow-up visit/End of study) throughout the trial. Every visit has ± 03 days of the window. The study duration spanned 31 weeks (03 Weeks for Screening/24 Weeks of Treatment Intervention, and 04 Weeks of Safety Follow-up).

Caregivers were advised to administer the tablets once a day in the morning. A simple traditional grinding device was provided to all families so tablets could be ground and mixed into cold food (yogurt, applesauce, fruit juice, shakes, *etc*.). Most families provided tablets in this manner. All children completed baseline measures (described below) and provided urine samples during the same 2-week screening window.

The study employed various assessments and procedures, including validated tools as follows:

### 4.1 Diagnostic and Statistical Manual of Mental Disorders (DSM-V)

Developed by the American Psychiatric Association, it outlines the standard diagnostic criteria for ASD. Diagnosis requires the presence of persistent deficits in social communication and interaction alongside restricted, repetitive patterns of behavior, interests, or activities. These symptoms must manifest in early childhood and cause clinically significant impairment in functioning. Specific criteria include deficits in social reciprocity, nonverbal communication, relationships, restricted interests, repetitive behaviors, and sensory sensitivities. Symptoms should not be better explained by intellectual disability or other developmental delays. Clinicians utilize these criteria, along with standardized assessment tools, to diagnose ASD and determine symptom severity, providing a standardized framework for research and treatment planning in ASD [21].

### 4.2 Social Responsiveness Scale (SRS-2)

A standardized assessment tool is used to evaluate social behavior in individuals with ASD and related conditions. Administered by qualified, trained professionals, the SRS-2 involves completing a questionnaire rating the frequency and severity of social behaviors observed over a specified period by parents, teachers, or caregivers. This assessment scale comprises 65 questionnaire including social awareness, social cognition, social communication, social motivation, autistic mannerisms, and baseline raw SRS total score. Responses are scored on a Likert scale, with higher scores indicating more significant social impairment. Total and subscale scores provide measures of social responsiveness and specific aspects of social behavior. The SRS-2 aids in diagnosis, treatment planning, and outcome measurement by identifying social deficits, and evaluating treatment effectiveness [22].

### 4.3 Childhood Autism Rating Scale (CARS)

It involves structured observations of the child's behavior and interactions. CARS assessment evaluates various behavior domains, including social interactions, communication skills, repetitive behaviors, and sensory sensitivities. Each behavior is rated based on severity and frequency, with higher scores indicating more significant impairment. The total score on the CARS provides an overall measure of autism severity, aiding in diagnostic decision-making and treatment planning [23].

### 4.4 Adaptive Behavior Scale (ABS)

ABS involves structured observations and interviews with individuals and their caregivers to assess their ability to navigate everyday tasks and social situations. The ABS scale primarily measures adaptive behavior in individuals with cognitive impairments and developmental disabilities. The scale measures adaptive behaviors related to communication, socialization, daily living, and motor skills. Responses are scored based on the individual's performance compared to age-appropriate expectations, with higher scores indicating better adaptive functioning. The ABS is valuable for diagnosing developmental disorders, planning interventions, and monitoring progress. Its comprehensive assessment of adaptive behaviors provides insights into an individual's strengths and areas needing support, facilitating tailored interventions to improve overall functioning and quality of life [24].

### 4.5 Clinical Global Impressions (CGI) Scale

CGI involves a clinician's subjective rating based on their observations and interactions with the patient. It consists of two main components: the CGI-Severity (CGI-S) scale and the CGI-Improvement (CGI-I) scale. The CGI-S assesses the severity of the patient's illness at the time of assessment, ranging from 1 (not at all ill) to 7 (severely ill). The CGI-I evaluates the patient's improvement or change in symptoms since the initiation of treatment, ranging from 1 (very much improved) to 7 (very much worse). The CGI provides a brief, standardized method for clinicians to communicate their clinical judgment regarding the patient's condition, allowing for easy comparison across different patients and treatment interventions. It is particularly valuable for tracking treatment response and guiding clinical decision-making [25].

All the study assessments (ADOS) were performed in compliance with the Schedule of assessments (Table **1**).

### 4.6 Statistical Analysis

Statistical analysis involved comparing the standard ASD treatment alone *versus* standard treatment combined with Altibrain**^®^** intervention using appropriate methods. Primary outcomes included change in core ASD symptoms measured by ADOS and safety assessments. Descriptive statistics, t-tests, *p*-value, and regression models were utilized to determine the significance of observed differences. Intent-to-treat analysis was employed to ensure the robustness of the findings.

## 5 RESULTS

A total of 120 participants were recruited, with 60 participants randomly assigned to the Standard ASD Treatment group and 60 to the Altibrain**^®^**+ Standard ASD Treatment group. The mean age of participants was 9.5 years (SD = 2.3), and the gender distribution was comparable between groups. Out of this, 71 were male and 49 were female as described in Fig. (**1**).

### 5.1 Diagnostic and Statistical Manual of Mental Disorders (DSM-V) Scale

**Fig. (2):**
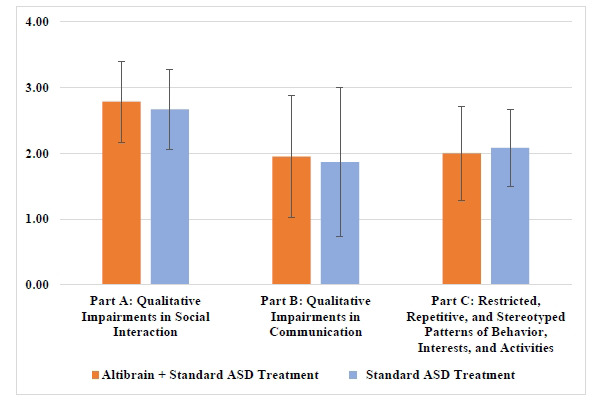
Mean scores in part A, B & C of the DSM-V criteria of ASD at baseline.

Comparing the Altibrain**^®^** + Standard ASD Treatment group with the Standard ASD Treatment group, the mean score for qualitative deficits in social interaction (Part A) was 2.78 (SD = 0.61) for both groups, indicating that Altibrain**^®^**significant effect. Comparable outcomes in communication impairments were seen between the Altibrain**^®^** + Standard ASD Treatment group (mean score of 1.95) and the Standard ASD Treatment group (1.87, SD = 1.13) in Part B, which measures qualitative impairments in communication. Part C of the test looked at confined, repetitive, and stereotypical behavior patterns, interests, and activities; this group scored 2.00 (SD = 0.71), while the Standard ASD Treatment group scored 2.08 (SD = 0.59). The results shows that there was no such statistical difference in both groups at baseline (Fig. **2**).

### 5.2 Social Responsive Scale-2 (SRS-2)

**Fig. (3):**
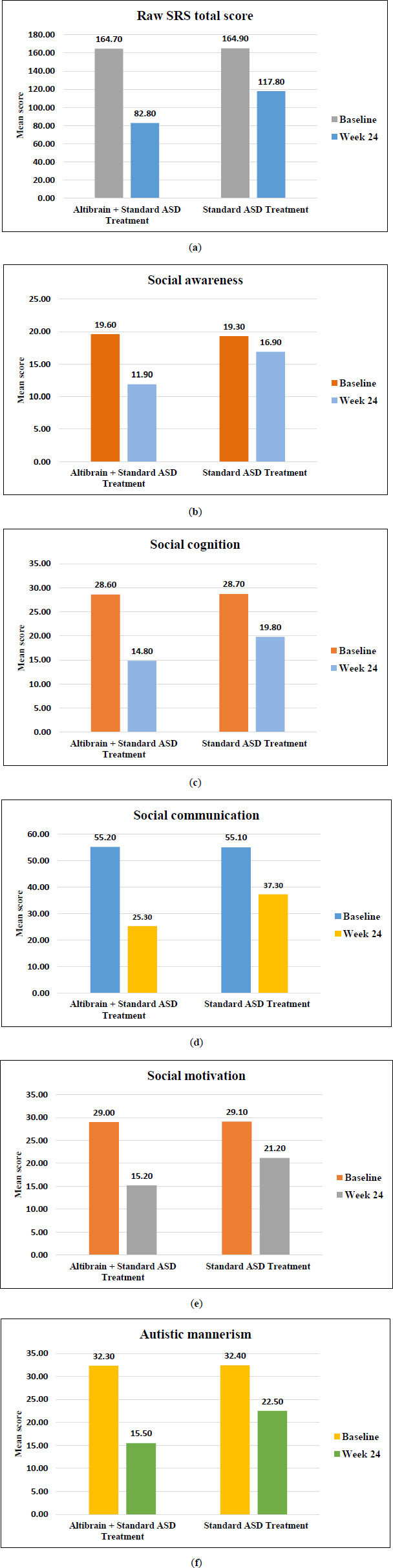
(**a**) Mean change in SRS-2 total score, (**b**) Mean change in SRS-2 social awareness score, (**c**) Mean change in SRS-2 social cognition score, (**d**) Mean change in SRS-2 social communication score, (**e**) Mean change in SRS-2 social motivation score, (**f**) Mean change in SRS-2 autistic mannerism score.

There is strong evidence that combining Altibrain**^®^** with traditional therapies for ASD improves social functioning more than conventional therapies for ASD alone. At the beginning (baseline) of the trial, ASD patients showed more severe symptoms than those in the usual treatment group on a number of scales measuring social awareness, cognition, communication, and motivation. However, after 24 weeks of treatment, those in the Altibrain**^®^** + Standard ASD Treatment group showed significant gains in social functioning, as indicated by lower scores on these measures (Figs. **3a**-**3f**).

When compared to the Altibrain**^®^** + conventional ASD treatment group, the baseline scores of the conventional ASD treatment group were higher *i.e*. higher SRS-2 scores. Significantly, when comparing the two groups' results at the end of the treatment period (24-week), those in the Altibrain**^®^** + Standard ASD Treatment group performed better and showed reduction in SRS-2 scores.

The results displayed in Table **2** show that incorporating Altibrain**^®^** into a treatment plan for ASD improves the efficacy of that plan in reducing social deficits. While all groups improved, the deficiencies in social functioning were more significantly reduced in the Altibrain**^®^** + Standard ASD Treatment group.

Results indicated in Table **2** showed notable improvements in social awareness, cognition, communication, motivation, and reduction in autistic mannerisms in both groups from baseline to week 24. However, the Altibrain**^®^** + Standard ASD Treatment group showed more significant improvements across all measured domains than the Standard ASD Treatment group alone. These improvements are reflected in both raw SRS total scores and T-scores, suggesting the potential benefit of incorporating Altibrain**^®^** intervention alongside standard ASD treatment for enhancing social functioning in individuals with ASD.

### 5.3 Change in Childhood Autism Rating Scale (CARS)

**Fig. (4):**
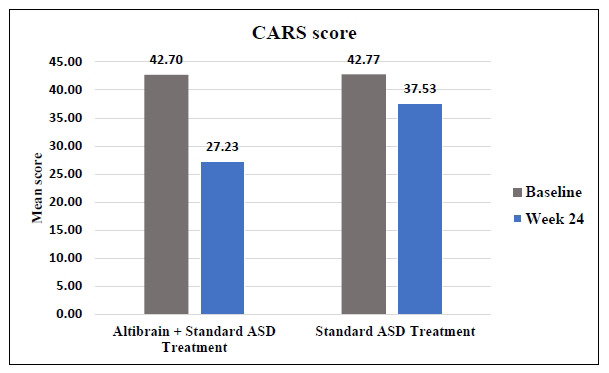
Change in Childhood Autism Rating Scale (CARS): Specific behaviors and characteristics associated with ASD.

The baseline mean scores for assessing the following aspects *i.e*. Relating to People, Emotional Response, Imitation, Body Use, Object Use, Adaptation to Change, Listening Response, Taste, Smell, Touch, Visual Response, Fear or Nervousness, Verbal Communication, and activity levels were similar between the Altibrain**^®^** + Standard ASD Treatment group and the Standard ASD Treatment group. There were no initial significant differences at baseline between the Altibrain**^®^** + Standard ASD Treatment group and the Standard ASD Treatment group (Total Score, 42.70 for Altibrain**^®^** + Standard ASD Treatment; 42.77 for Standard ASD Treatment; *p* = 0.9610). In addition, there was not much difference between the two groups' standard deviations, indicating a similar amount of internal variation at baseline. There was slight significant reduction in mean CARS score in Standard ASD treatment group at week-24 (Total score-37.53) but there was more significant defference observed in Altibrain^®^ + Standard ASD Treatment group at week-24 (Total score-27.23) (Fig. **4**).

### 5.4 Adaptive Behaviour Scale (ABS)

**Fig. (5):**
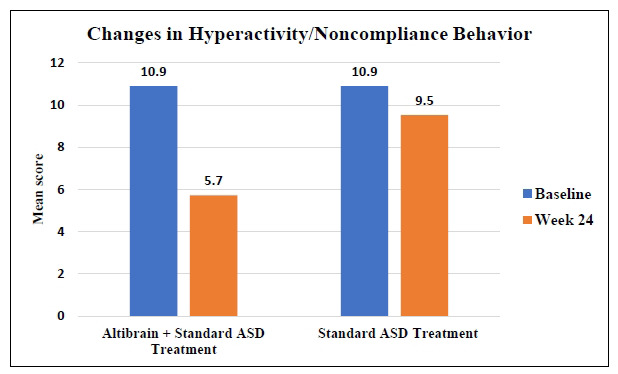
Comparative analysis of changes in hyperactivity/noncompliance behaviour with Altibrain^®^ + standard ASD treatment *vs*. standard ASD treatment.

Hyperactivity/Noncompliance symptoms were significantly reduced in the Altibrain**^®^** + Standard ASD Treatment group after 24 weeks of treatment, with the mean score dropping from 10.9 to 5.7. On the other hand, the mean score decreased from 10.9 to 9.5 in the Standard ASD Treatment group (Fig. **5**). These results indicate that the combination of Altibrain**^®^** and standard ASD treatment led to significant improvement (*p <* 0.0001) in Hyperactivity/Noncompliance symptoms than standard treatment alone, suggesting the potential superiority of the combined therapy in addressing this particular aspect of ASD symptomatology.

**Fig. (6):**
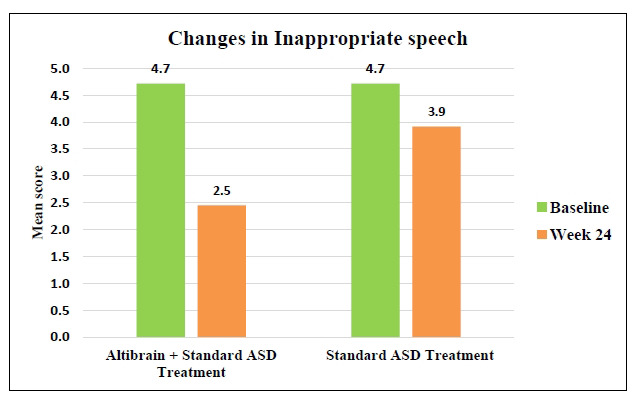
Comparative analysis of changes in inappropriate speech behaviour with Altibrain^®^ + standard ASD treatment *vs*. standard ASD treatment.

The average Inappropriate Speech symptom score decreased from 4.7 to 2.5 after 24 weeks of treatment in the Altibrain**^®^** + Standard ASD Treatment group (Fig. **6**). The standard ASD treatment group, in contrast, saw a less striking decrease, with the mean score going from 4.7 to 3.9 (*p <* 0.0001). These findings suggest that when addressing Inappropriate Speech symptoms, the combination of Altibrain**^®^** and standard ASD treatment is superior to standard treatment alone, indicating that the combined therapy may be more effective overall.

**Fig. (7):**
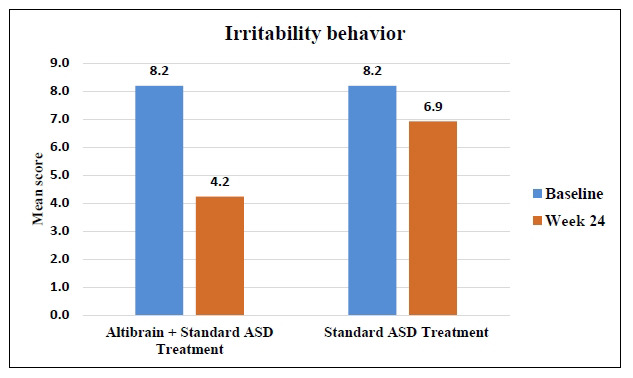
Comparative analysis of changes in irritability behaviour with Altibrain^®^ + Standard ASD treatment *vs*. standard ASD treatment.

The baseline and 24-week Irritability scores for the Altibrain**^®^** + Standard ASD Treatment and Standard ASD Treatment groups show significant differences in therapy response. Irritability symptoms were similar in both groups at baseline (8.2; *p <* 0.0001). With a mean score of 4.2, Altibrain**^®^** + Standard ASD Treatment reduced irritability symptoms after 24 weeks. The Standard ASD Treatment group also decreased slightly, with a mean score of 6.9. Irritability symptoms decreased much more with Altibrain**^®^** + Standard ASD Treatment than with standard treatment alone (Fig. **7**).

**Fig. (8):**
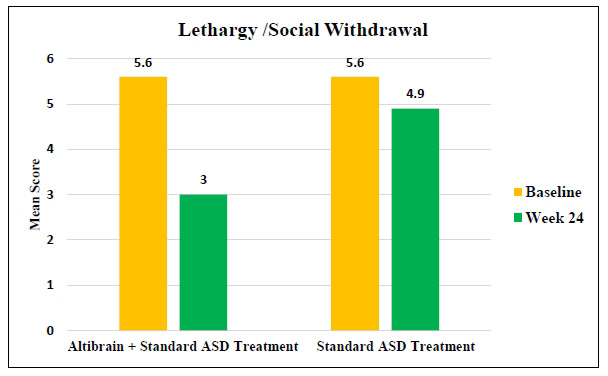
Comparative analysis of changes in lethargy/social withdrawal behaviour with Altibrain^®^ + standard ASD treatment *vs*. standard ASD treatment.

The Altibrain**^®^** + Standard ASD Treatment group showed a significant improvement in Lethargy/Social Withdrawal symptoms, with the mean score dropping from 5.6 to 3.0 after 24 weeks of treatment (Fig. **8**). Mean scores dropped from 5.6 to 4.9 (*p <* 0.0001) in the Standard ASD Treatment group. These results highlight the potential superiority of the Altibrain**^®^** + Standard ASD Treatment combination in addressing the symptoms of Lethargy/Social Withdrawal in individuals with ASD, as compared to individuals receiving only standard treatment.

**Fig. (9):**
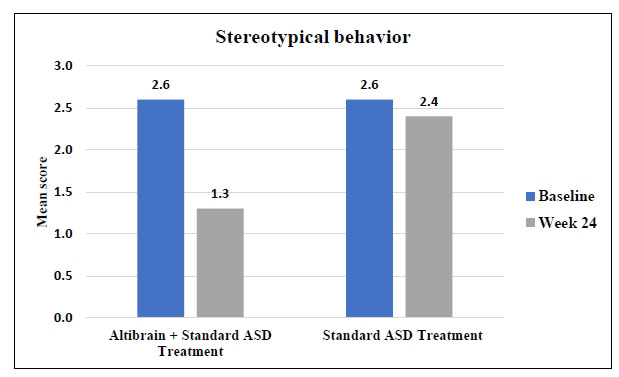
Comparative analysis of changes in stereotypical behaviour with Altibrain^®^ + standard ASD treatment *vs*. standard ASD treatment.

The average score for Stereotypical Behaviour decreased from 2.6 to 1.3 (*p* < 0.0001) after 24 weeks of treatment in the Altibrain**^®^** + Standard ASD Treatment group. The standard ASD treatment group, in contrast, also showed improvement, with a decrease in mean score from 2.6 to 2.4. These findings suggest that the combination of Altibrain**^®^** and standard ASD treatment is superior to the standard treatment alone in alleviating the symptoms of stereotypic behaviour, which is a hallmark of the disorder (Fig. **9**).

**Fig. (10):**
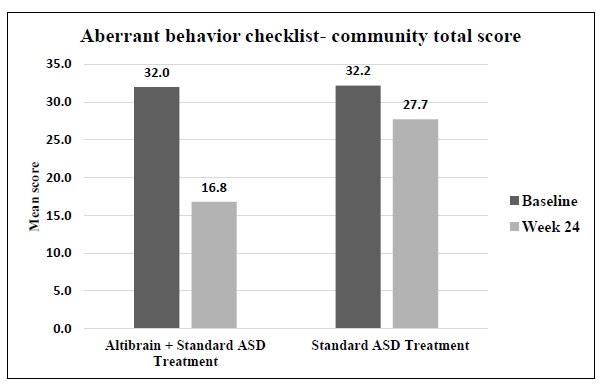
Comparative analysis of changes in aberrant behaviour checklist - community score with Altibrain^®^ + standard ASD treatment *vs*. standard ASD treatment.

The average score for abnormal behaviours decreased from 32.0 to 16.8 (*p <* 0.0001) after 24 weeks of treatment in the Altibrain**^®^** + Standard ASD Treatment group. The average score for the standard ASD treatment group decreased from 32.2 to 27.7; but not to the same extent. These findings suggest that compared to standard treatment alone, the combination of Altibrain**^®^** and standard ASD treatment led to a significantly more significant improvement in aberrant behaviours, highlighting the potential superiority of the combined therapy in addressing this particular aspect of ASD symptomatology (Fig. **10**).

### 5.5 Clinical Global Impression (CGI) Scale

**Fig. (11):**
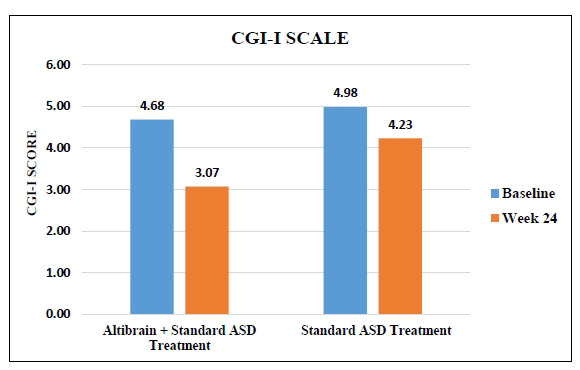
Comparative analysis of changes in Part-I severity of CGI with Altibrain^®^ + standard ASD treatment *vs*. standard ASD treatment.

Significant differences in treatment response were found between the Altibrain**^®^** + Standard ASD Treatment group and the Standard ASD Treatment group, as measured by changes in Clinical Global Impression (CGI) Part-I Severity. Both groups' mean CGI Severity scores were similar at baseline. Still, after 24 weeks of treatment, those in the Altibrain**^®^** + Standard ASD Treatment group showed a significant reduction in clinical severity, with a score of 3.07 (*p <* 0.0001). Those in the Standard ASD Treatment group also improved, but to a lesser extent, with a score of 4.23. Adding Altibrain**^®^** to normal ASD treatment resulted in a much more significant reduction in clinical severity, highlighting the potential superiority of the combined therapy in addressing the severity of ASD symptoms as assessed by CGI Part-I Severity (Fig. **11**).

### 5.6 Adverse Event

The adverse event data from the study comparing Altibrain**^®^** + Standard ASD Treatment with Standard ASD Treatment alone reveals notable differences in the occurrence and severity of various adverse events. This summarizes the number of occurrences and severity of each adverse event in both treatment groups (Table **3**).

The results indicate that certain adverse events such as gastric discomfort or irritation, heartburn, and hypothermia were more prevalent in the Altibrain**^®^** + Standard ASD Treatment group compared to the Standard ASD Treatment group. Conversely, insomnia was notably higher in the Standard ASD Treatment group. Sedation and anxiety were reported in both groups, albeit with slightly higher occurrences in the Standard ASD Treatment group. The majority of adverse events were categorized as mild, with insomnia being the most frequently reported moderate adverse event in both groups. These findings suggest potential differences in the safety profiles between the two treatment regimens, warranting further investigation and consideration in clinical decision-making.

## 6 DISCUSSION

The study provides a comprehensive analysis of the potential efficacy and safety of Altibrain^®^ + Standard ASD Treatment as a treatment for ASD when compared to Standard ASD Treatment alone. The research design was carefully devised to encompass a wide range of dimensions pertaining to ASD, such as fundamental symptoms, social communication abilities, repetitive behaviors, and overall functional enhancement. The study attempted to determine the effectiveness of Altibrain^®^+ Standard ASD Treatment as an additional treatment for ASD by comparing it to the standard ASD treatment alone. This was done by dividing the participants into two groups, one receiving Altibrain^®^ along with the standard treatment and the other receiving only the standard treatment.

The results of our study demonstrate a substantial reduction in the primary symptoms of ASD among the participants who received treatment with Altibrain^®^ + Standard ASD Treatment. This improvement was quantified using the ADOS showing considerable clinical advancement. The findings of this study are consistent with prior research conducted on animals (preclinical) and humans (clinical), which also show the effectiveness of Altibrain^®^ + Standard ASD Treatment in reducing the main symptoms of ASD [26-28]. Furthermore, there were notable advancements in social communication abilities, as evidenced by improved scores on the Social Responsiveness Scale (SRS) among individuals who received Altibrain^®^+ Standard ASD Treatment. This highlights the crucial significance of addressing this aspect to achieve overall functional enhancement and well-being in people with ASD [29-33].

Furthermore, Altibrain**^®^**+ Standard ASD Treatment exhibited the ability to reduce repetitive behaviors, as evidenced by significant improvements in Adaptive Behavior Scale (ABS). These findings are consistent with previous studies using animal models [34] and small-scale clinical trials [33], highlighting the multifaceted potential of sulforaphane in addressing the complex symptomatology of ASD [35, 36].

Notably, the study also confirms the favorable safety and tolerability profile of sulforaphane as indicated by the absence of significant variations in adverse events between the two treatment groups. These results are in line with previous investigations and suggest that sulforaphane may offer a therapeutic option with good tolerability for individuals with ASD [37, 38].

While our findings hold promise for the clinical application of Altibrain**^®^** as a comprehensive intervention approach for ASD, several limitations must be acknowledged. The relatively short duration of the trial may provide only a limited perspective on the long-term effects of Altibrain**^®^**. Additionally, although the sample size provided sufficient statistical power, a more extensive and diverse participant pool could enhance the generalizability of the results. Future research endeavors should also delve deeper into the fundamental neurobiological mechanisms underlying the effects of Altibrain**^®^**, utilizing advanced imaging techniques or biomarker analyses to elucidate its impact on ASD symptomatology.

This study highlights the promising potential of Altibrain**^®^** as an adjunctive intervention for ASD, demonstrating its efficacy in reducing core symptoms, improving social communication, and addressing repetitive behaviors [30, 31]. While the findings are encouraging, further research is warranted to confirm and expand upon these results, including longer-term studies with larger cohorts and a more comprehensive exploration of underlying mechanisms [39]. Nonetheless, the diverse and multifaceted effects of Altibrain**^®^** signal optimism for enhancing the well-being of individuals on the ASD spectrum and their families, marking a significant contribution to the advancement of therapeutic approaches for ASD [40-42] (See supplementary figures and tables).

## 7 LIMITATIONS OF THE STUDY

While this study contributes valuable insights into the potential efficacy and safety of Altibrain**^®^** as an intervention for ASD, several limitations warrant consideration. Firstly, the study's relatively short duration of 24 weeks may not capture the full spectrum of Altibrain**^®^**'s long-term effects. A more extended follow-up period would provide a more comprehensive understanding of the treatment's durability and sustained impact. Secondly, the sample size, though adequate for the current analyses, may limit the generalizability of the findings to broader ASD populations. A larger and more diverse cohort could enhance the robustness of the results and allow for subgroup analyses based on age, severity, and other relevant factors. Additionally, the study predominantly focused on behavioral outcomes, and while these are crucial, integrating neurobiological investigations such as neuroimaging [43-47] or biomarker analyses [40, 41] could provide deeper insights into the underlying mechanisms of Altibrain's effects.

## 8 FUTURE DIRECTIONS

Building upon the foundations established by this study, future research directions are essential to further advance our understanding of Altibrain's**^®^** potential as an intervention for ASD. Longitudinal studies with extended follow-up periods are warranted to investigate the long-term benefits and potential side effects of Altibrain**^®^** treatment. A more expansive and diverse participant pool, including individuals from various demographics and geographic regions, would enhance the generalizability of findings and allow for more comprehensive subgroup analyses. Furthermore, deeper investigations into the neurobiological mechanisms of Altibrain**^®^**'s effects through advanced imaging techniques, neurophysiological assessments, and biomarker studies are recommended. These efforts could unveil the precise pathways through which Altibrain**^®^** modulates ASD symptomatology and potentially inform personalized treatment strategies. Additionally, exploring Altibrain**^®^**'s effects in combination with existing interventions or therapies, such as behavioral interventions or other pharmacological agents, could provide a more holistic approach to ASD management. Overall, a concerted interdisciplinary effort involving clinicians, researchers, and individuals with ASD and their families is imperative to fully realize the clinical potential of Altibrain**^®^** and improve the quality of life for those living with ASD.

## CONCLUSION

This study offers compelling evidence of Altibrain's**^®^** potential as an intervention for ASD patients, highlighted by its significant impact on core ASD symptoms, social communication abilities, and repetitive behaviors. The results are consistent with earlier studies conducted on animals and humans, confirming the effectiveness of Altibrain's^®^ in improving several aspects of ASD symptoms. Although there are limitations to the study and more research is needed, these findings highlight the potential clinical significance of Altibrain's^®^ and contribute to our knowledge of effective treatments for ASD. This offers hope for improving the well-being and quality of life of individuals with ASD and their families.

## Figures and Tables

**Fig. (1):**
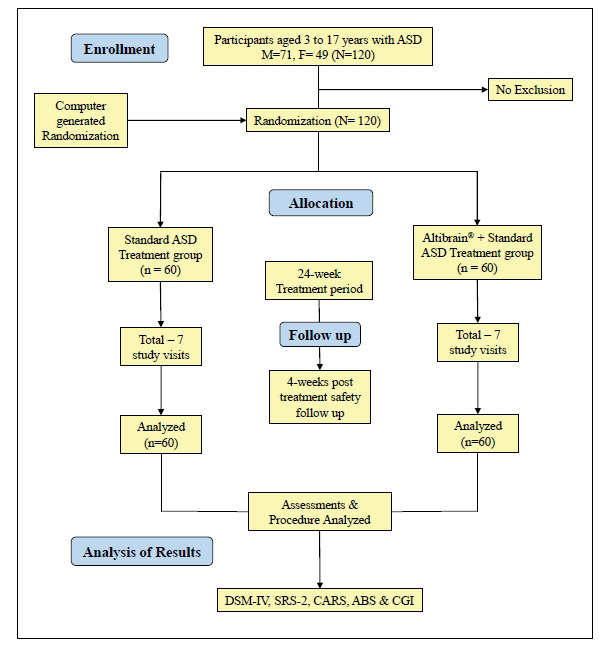
Flow diagram of participants studied in the trial.

**Table 1:** Schedule of assessments.

**ADOS (Autism Diagnostic Observation Schedule)**	**Visit 01**	**Visit 02**	**Visit 03**	**Visit 04**	**Visit 05**	**Visit 06**	**Visit 07**
	**Screening Visit (-21 Days)**	**Day 01/ Baseline Visit**	**Day 29/ Week 04**	**Day 85/ Week 12**	**Day 127/ Week 18**	**Day 169/Week 24/ End of Treatment**	**EOT + 28 Days/ Safety Follow-up Visit/End of Study**
Informed Consent	✓	-	-	-	-	-	-
Inclusion and Exclusion Criteria	✓	-	-	-	-	-	-
Demography	✓	-	-	-	-	-	-
Medical History	✓	-	-	-	-	-	-
Vital Signs	✓	✓	✓	✓	✓	✓	✓
Physical examination	✓	✓	✓	✓	✓	✓	✓
Randomization	-	✓	-	-	-	-	-
DSM-V criteria confirmation	✓	✓	-	-	-	-	-
Social Responsiveness Scale (SRS-2)	✓	✓	✓	✓	✓	✓	-
Childhood Autism Rating Scale (CARS)	✓	✓	✓	✓	✓	✓	-
Adaptive Behavior Scale (ABS)	✓	✓	✓	✓	✓	✓	-
Clinical Global Impressions (CGI) scale	✓	✓	✓	✓	✓	✓	-
Urine sample collection	✓	✓	✓	✓	✓	✓	-
Investigational Product (IP) Dispensation^1^	-	✓	✓	✓	✓	-	-
IP Accountability and Return	-	-	✓	✓	✓	✓	-
AE / SAE Recording^2^	-	✓	✓	✓	✓	✓	✓
Prior and Concomitant Medications	✓	-	-	-	-	-	-

**Table 2:** Comparison of SRS-2 scores in standard ASD treatment *versus* Altibrain^®^ + standard ASD treatment group from baseline to **week 24.**

**SRS-2**	**Standard ASD Treatment Group**	**Standard ASD Treatment Group**	**Altibrain^®^ + Standard ASD Treatment Group**	**Altibrain^®^ + Standard ASD Treatment Group**
	**Baseline**	**Week 24**	**Baseline**	**Week 24**
Social awareness	19.3 ± 2.42	16.93 ± 25.78	19.55 ± 2.51	11.93 ± 1.88
Social cognition	28.72 ± 2.6	19.75 ± 2.06	28.63 ± 2.6	14.75 ± 2.06
Social communication	55.08 ± 2.54	37.32 ± 2.97	55.2 ± 2.55	25.32 ± 2.84
Social motivation	29.08 ± 4.3	21.23 ± 2.55	29 ± 4.25	15.23 ± 2.28
Autistic mannerisms	32.37 ± 2.6	22.53 ± 2.57	32.27 ± 2.46	15.53 ± 2.28
Baseline raw SRS total score	164.9 ± 8.35	117.8 ± 6.04	164.65 ± 8	82.77 ± 4.51
SRS-2 T-Score	87 ± 1.58	86.96 ± 2.33	87.75 ± 1.49	72.72 ± 1.84

**Table 3:** Comparison of adverse events between Altibrain^®^ plus standard ASD treatment and standard ASD treatment alone.

**Adverse Event**	**Altibrain^®^ + Standard ASD Treatment**	**Standard ASD Treatment**	**Severity**
Hyperlipidemia	2	8	Mild
Sedation	5	11	Mild
Hyperglycemia	0	2	Mild
Insomnia	4	16	Moderate
Headache	2	7	Mild
Anxiety	3	11	Mild
Gastric discomfort or irritation	8	2	Moderate
Heartburn	7	1	Mild
Hypothermia	3	0	Mild
Fatigue	2	0	Mild

## Data Availability

All data generated or analyzed during this study are included in this published article.

## LIST OF ABBREVIATIONS

ABS: Adaptive Behavior Scale
ADOS: Autism Diagnostic Observation Schedule
ASD: Autism Spectrum Disorder
CARS: Childhood Autism Rating Scale
CGI: Clinical Global Impression
SRS-2: Social Responsive Scale-2

## STANDARDS OF REPORTING

CONSORT guidelines were followed.
