# Supplementary material for: Efficacy and Safety of Altibrain® as an Adjunctive Therapy for Autism Spectrum Disorder: An Open Label Trial Targeting Core Symptoms
Source: Curr Pharm Des. 2025 Jan 24;31(17):1388–401. doi: 10.2174/0113816128335544241210144541 (PMC12307958; doi:10.2174/0113816128335544241210144541)

Supplementary Material

Efficacy and Safety of Altibrain® as an Adjunctive Therapy for Autism Spectrum Disorder: A Randomized Controlled Trial Targeting Core Symptoms

Sidharth Mehan[1,*], Aakash Kumar[1], Prashant R. Utage[2], Anaita Hegde[3], Neeta Naik[4], Santosh Kondekar[5], Nandan Yardi[6], Neelu Desai[7], Debasis Panigrahi[8], Arijit Chattopadhyay[9], Sasmita Devi Agarwal[10], V.B. Gupta[11], Ankita Tiwari[12], Sai Chandar Reddy[13], Sandeep Saraf[14], Diptanshu Das[15], Mayank Detroja[16] and Charu Paliwal[17]

[1]Division of Neuroscience, Department of Pharmacology, ISF College of Pharmacy, Moga, Punjab, India (Affiliated to IK Gujral Punjab Technical University, Jalandhar, Punjab, 144603, India); [2]Pediatric Neurologist, Utage Child Development Centre, Hyderabad, India; [3]Pediatric Neurologist, SRCC Hospital, Mumbai, India; [4]Pediatric Neurologist, EN1 Neuroservices Pvt. Ltd, Mumbai, India; [5]Pediatric Neurologist, Aakar Clinic, Mumbai, India; [6]Pediatric Neurologist, Yardi Hospital, Pune, India; [7]Pediatric Neurologist, Hinduja Hospital, Mumbai, India; [8]Pediatric Neurologist, Ankur Hospital, Bhubaneshwar, India; [9]Developmental Pediatrician, Apollo Multispecialty Hospitals, Kolkata, India; [10]Pediatric Neurologist, Hi-Tech Medical College & Hospital - Bhubaneswar, India; [11]Pediatric Neurologist, Apollo Hospitals, Delhi, India; [12]Pediatric Neurologist, Lotus Hospital, Indore, India; [13]Pediatric Neurologist, Dr. Sai Chandar Neurocare, Warangal, India; [14]Pediatric Neurologist, Star Kids Hospital, Aurangabad, India; [15]Pediatric Neurologist, Institute of Neurodevelopment, Kolkata, India; [16]Pediatric Neurologist, Sahaj Child Neurology & Epilepsy Centre, Surat, India; [17]Director Operations and Medical Writing, RYT Lifesciences Pvt Ltd, Ahmedabad, Gujrat, India

1873-4286/25 © 2025 Bentham Science Publishers

Figure 1: Mean scores in Part A, B & C of the DSM-IV Criteria of ASD at Baseline

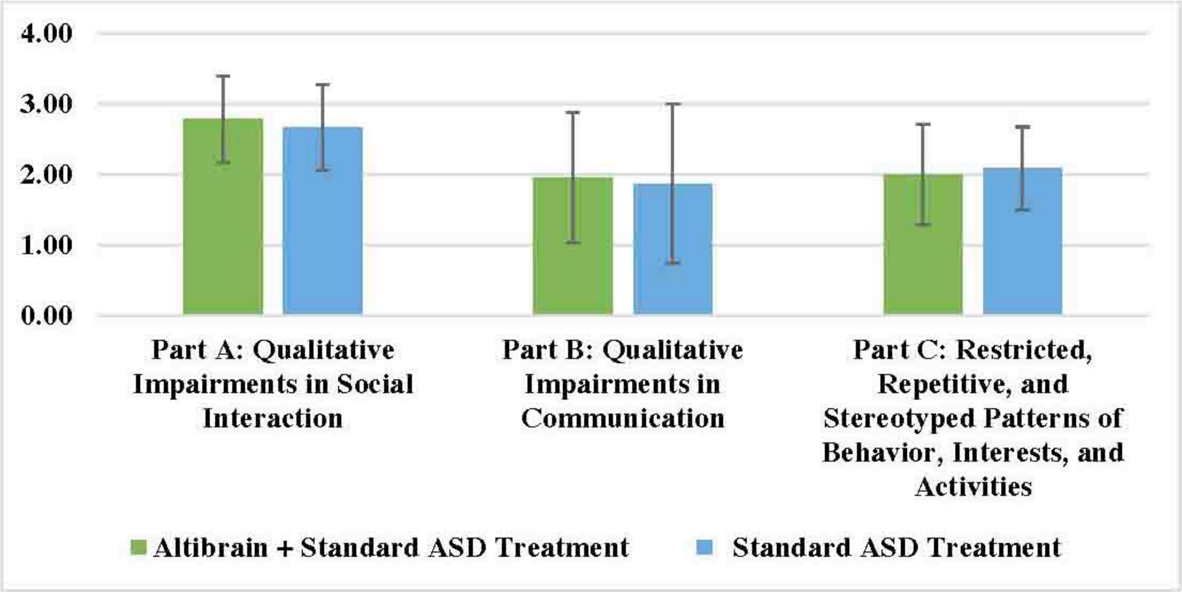

Figure 2a: Mean Change in SRS-2 Total Score

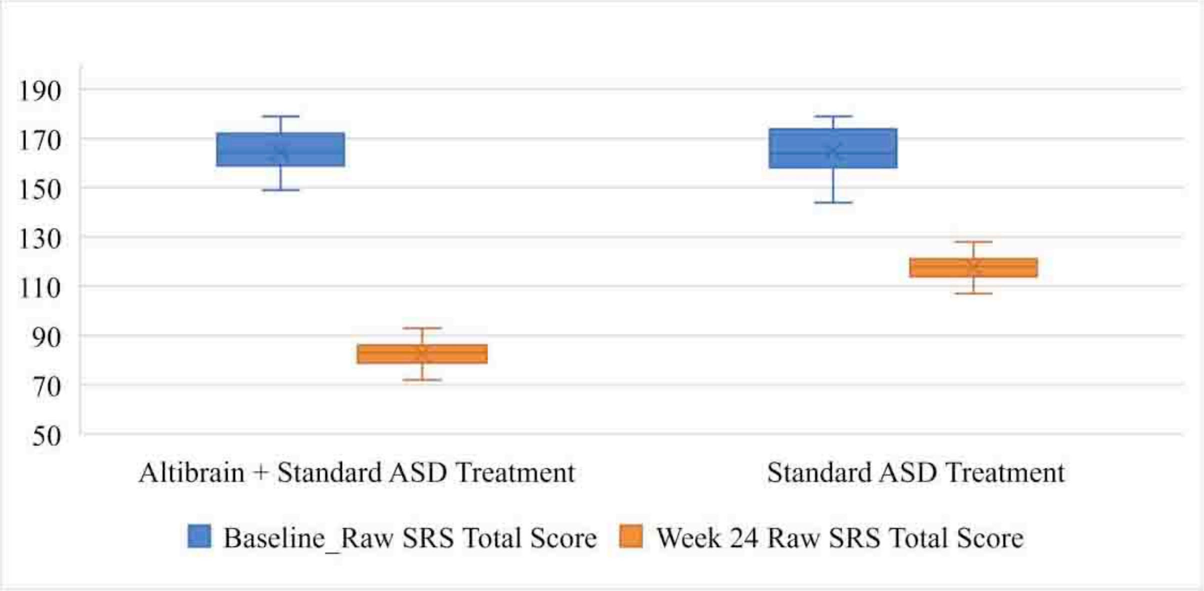

Figure 2b: Mean Change in SRS-2 Social Awareness Score

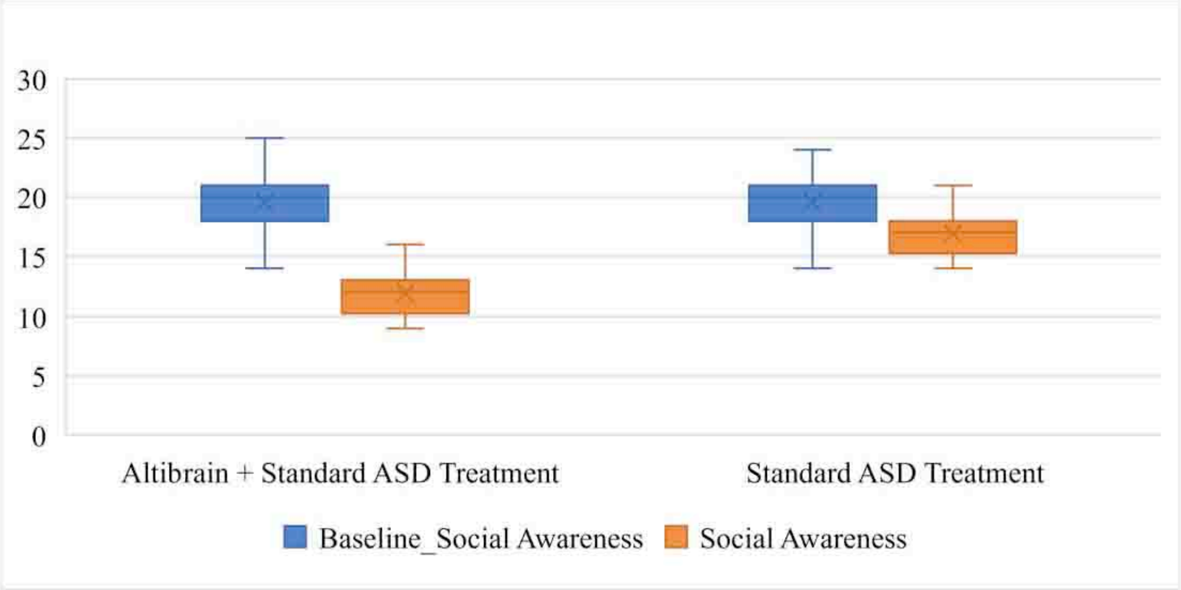

Figure 2c: Mean Change in SRS-2 Social Cognition Score

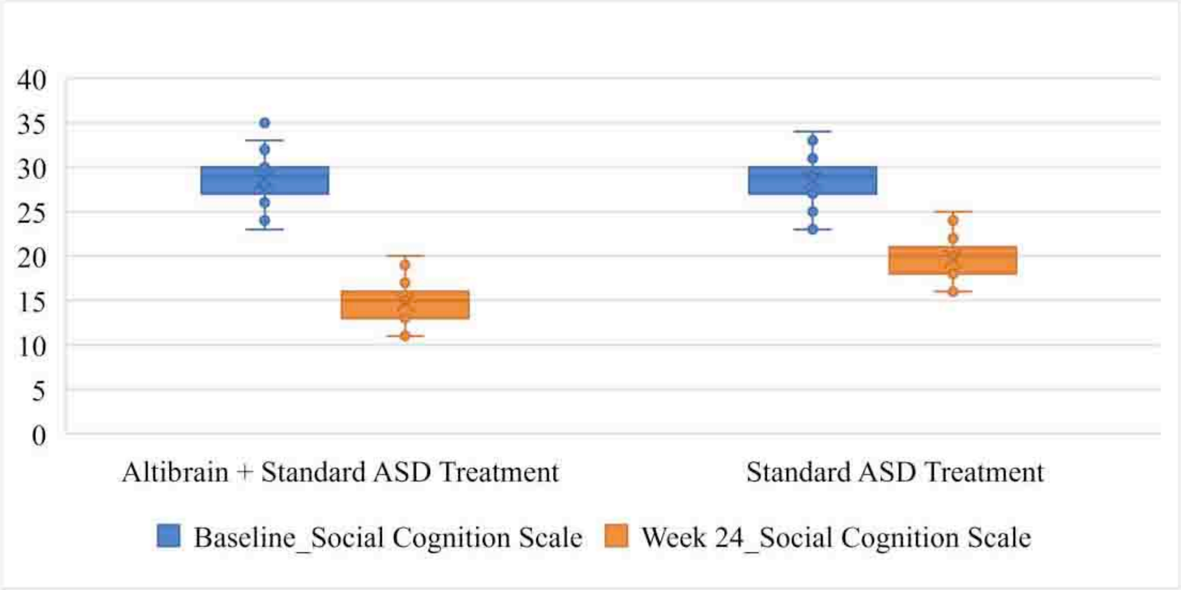

Figure 2d: Mean Change in SRS-2 Social Communication Score

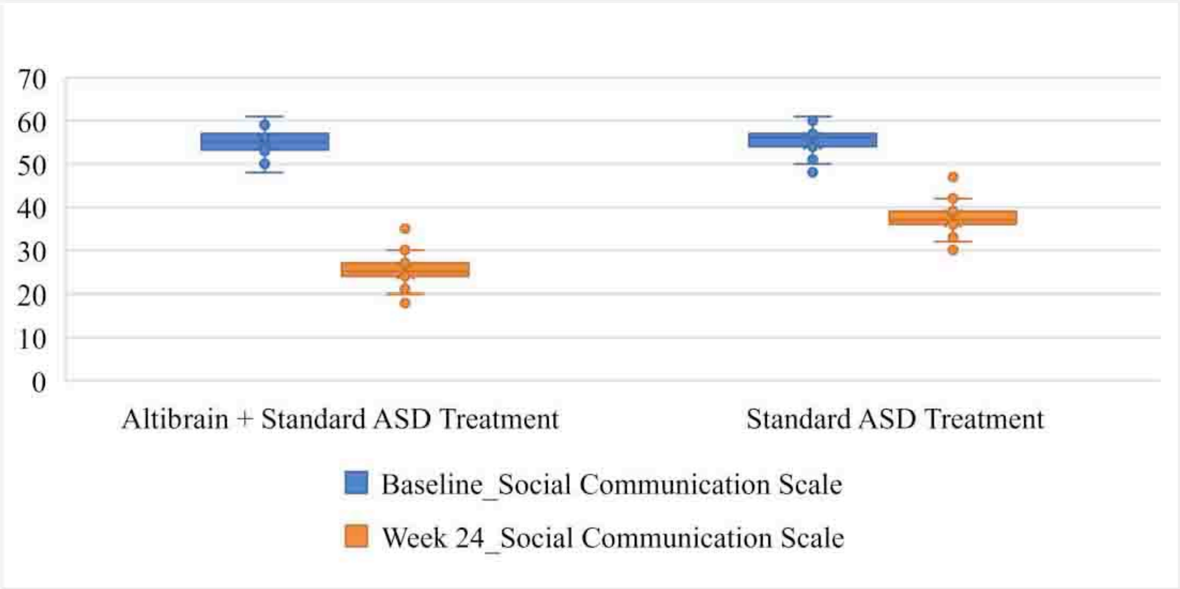

Figure 2e: Mean Change in SRS-2 Social Motivation Score

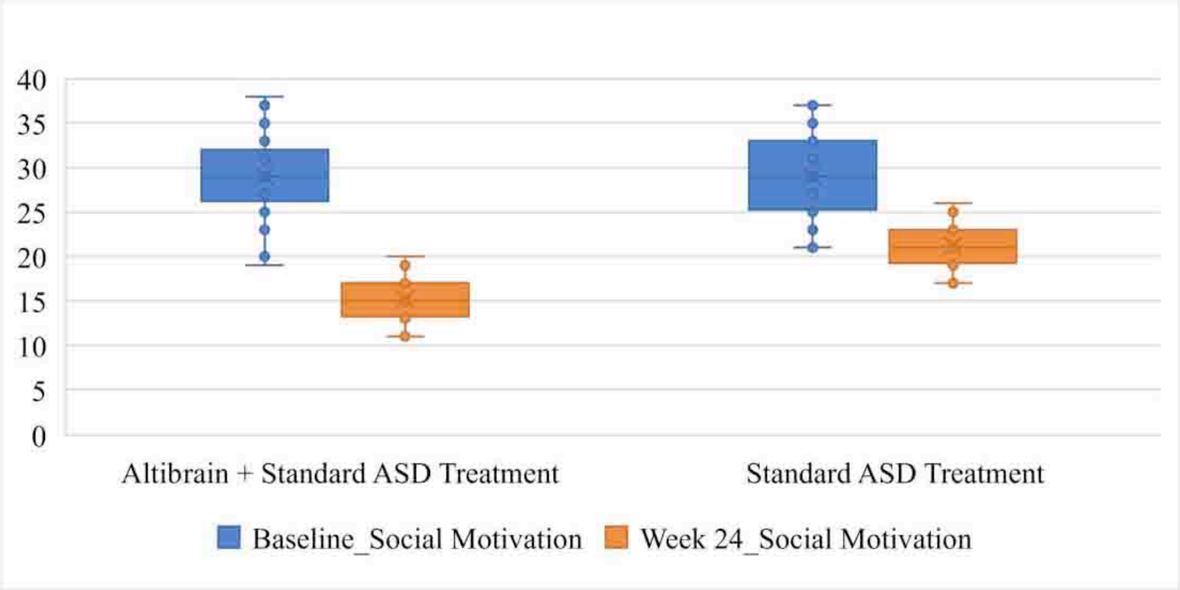

Figure 2f: Mean Change in SRS-2 Autistic Mannerisms Score

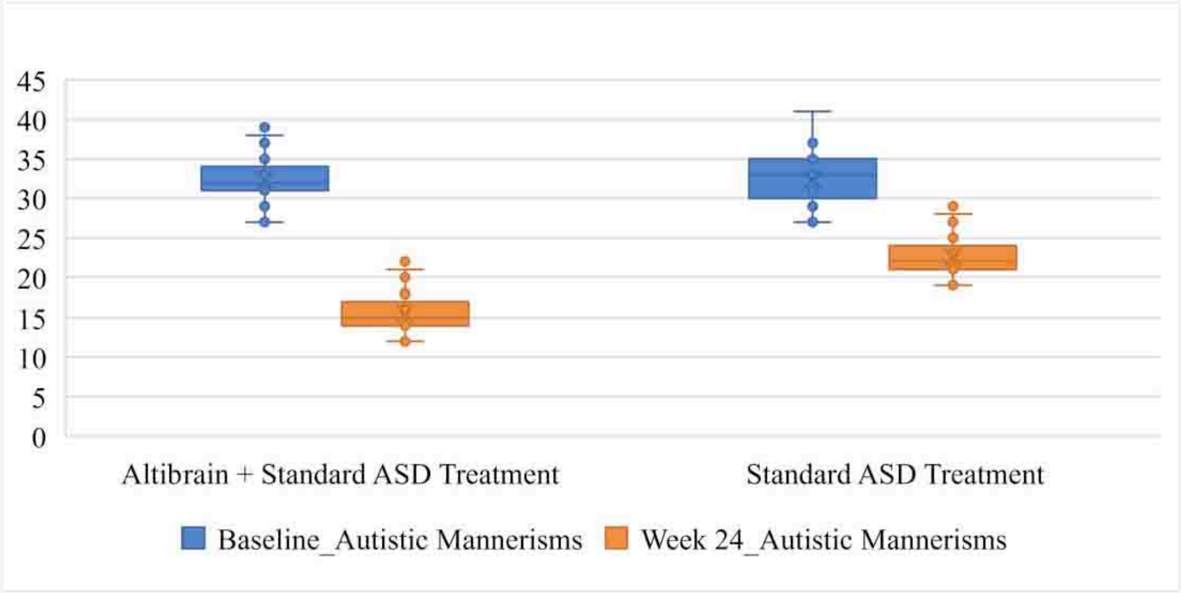

Figure 8: Change in Childhood Autism Rating Scale (CARS): Specific behaviours and characteristics associated with ASD.

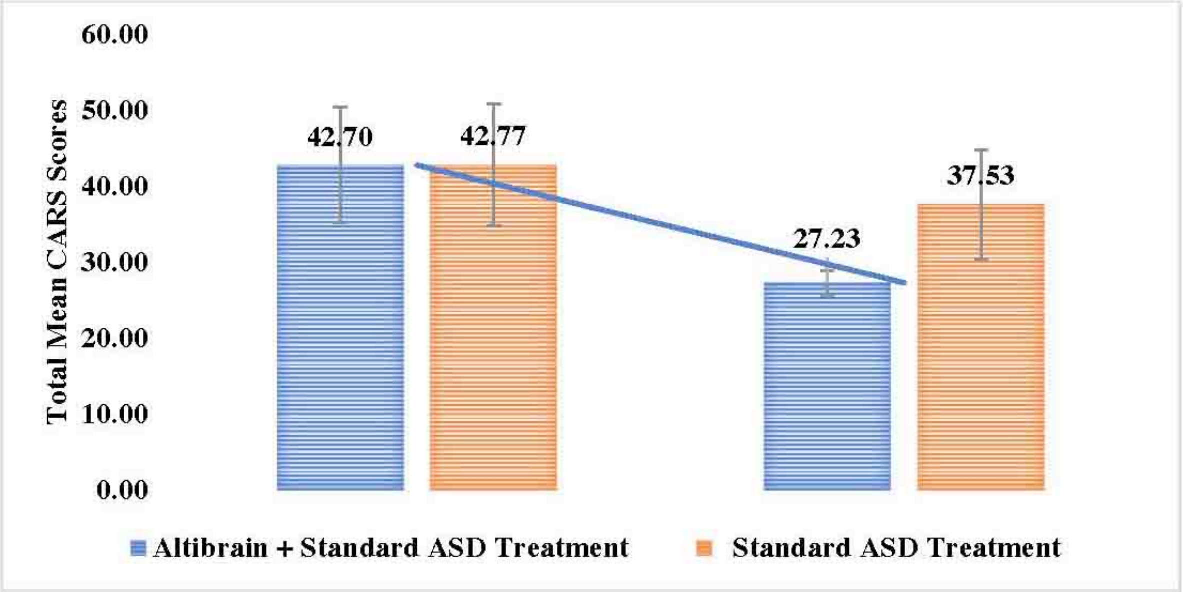

Figure 9: Comparative Analysis of Changes in Hyperactivity/Noncompliance Behaviour with Altibrain® + Standard ASD Treatment vs. Standard ASD Treatment

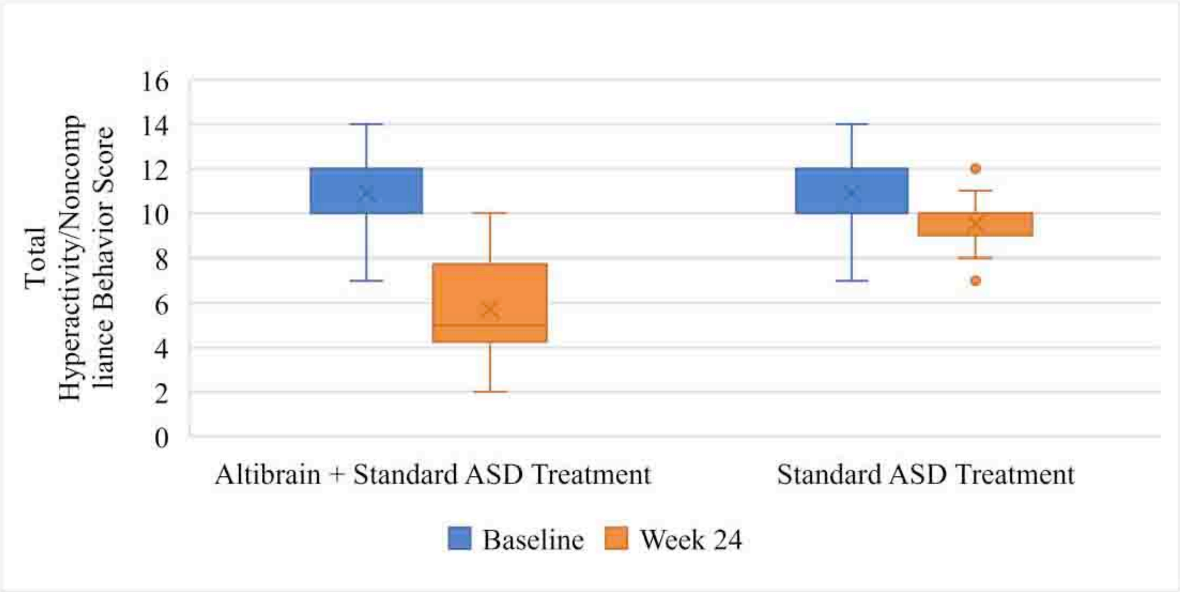

Figure 10: Comparative Analysis of Changes in Inappropriate Speech Behaviour with Altibrain® + Standard ASD Treatment vs. Standard ASD Treatment

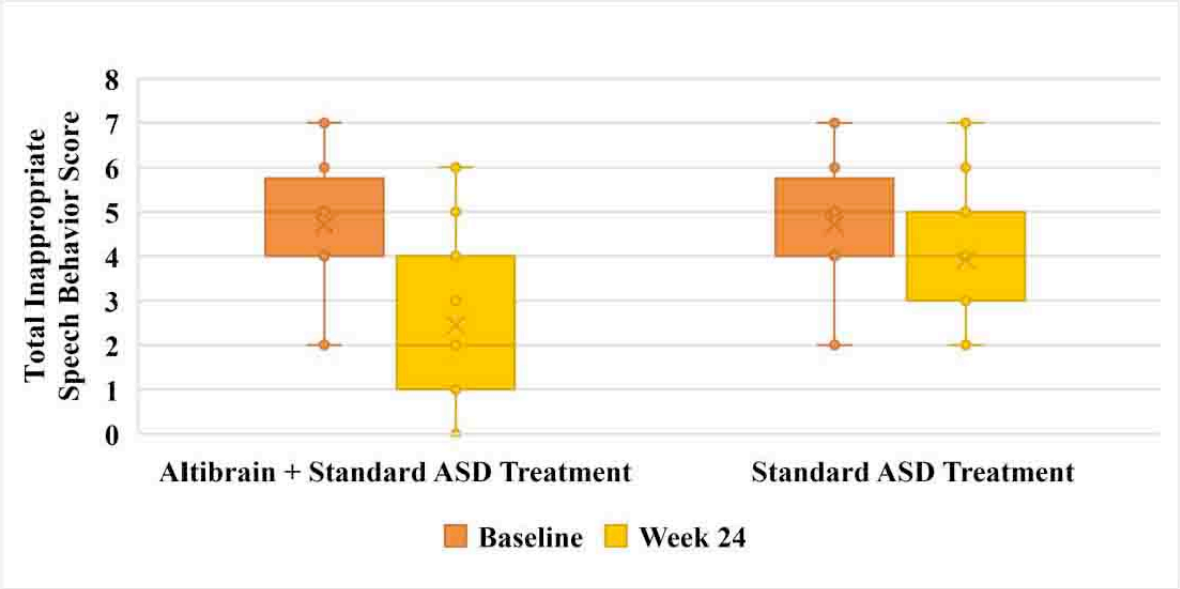

Figure 11: Comparative Analysis of Changes in Irritability Behaviour with Altibrain® + Standard ASD Treatment vs. Standard ASD Treatment

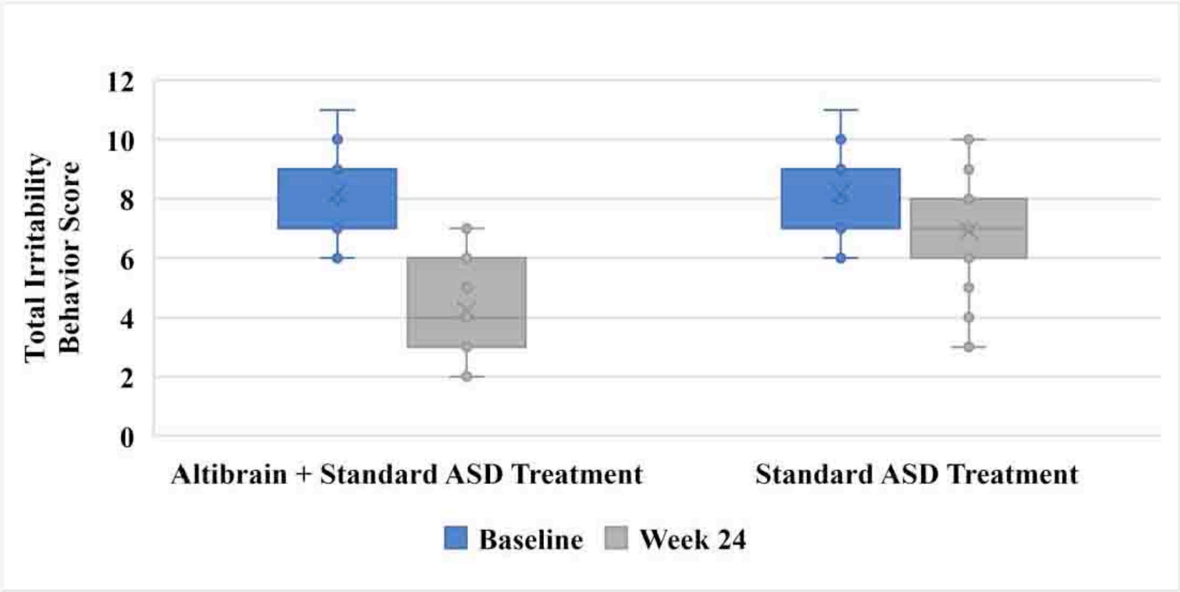

Figure 12: Comparative Analysis of Changes in Lethargy/Social Withdrawal Behaviour with Altibrain® + Standard ASD Treatment vs. Standard ASD Treatment

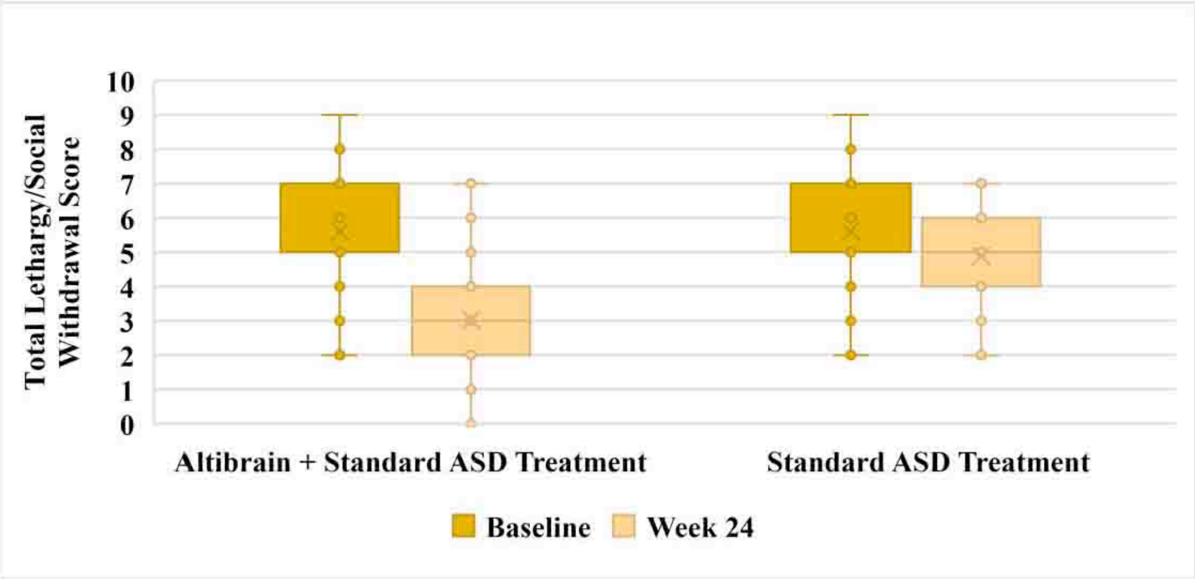

Figure 13: Comparative Analysis of Changes in Stereotypic Behaviour with Altibrain® + Standard ASD Treatment vs. Standard ASD Treatment

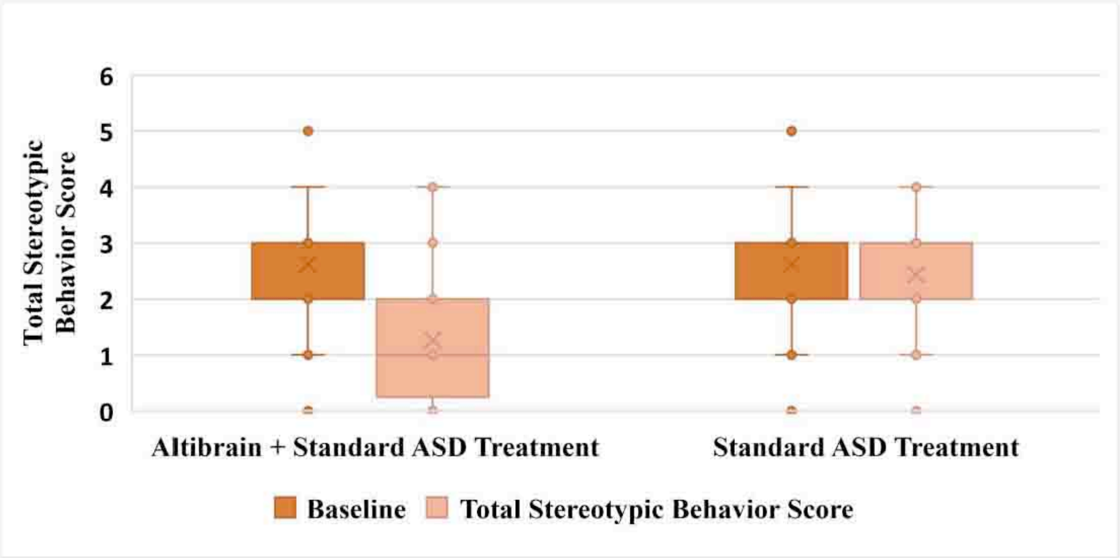

Figure 14: Comparative Analysis of Changes in Aberrant Behaviour Checklist - Community Score with Altibrain® + Standard ASD Treatment vs. Standard ASD Treatment

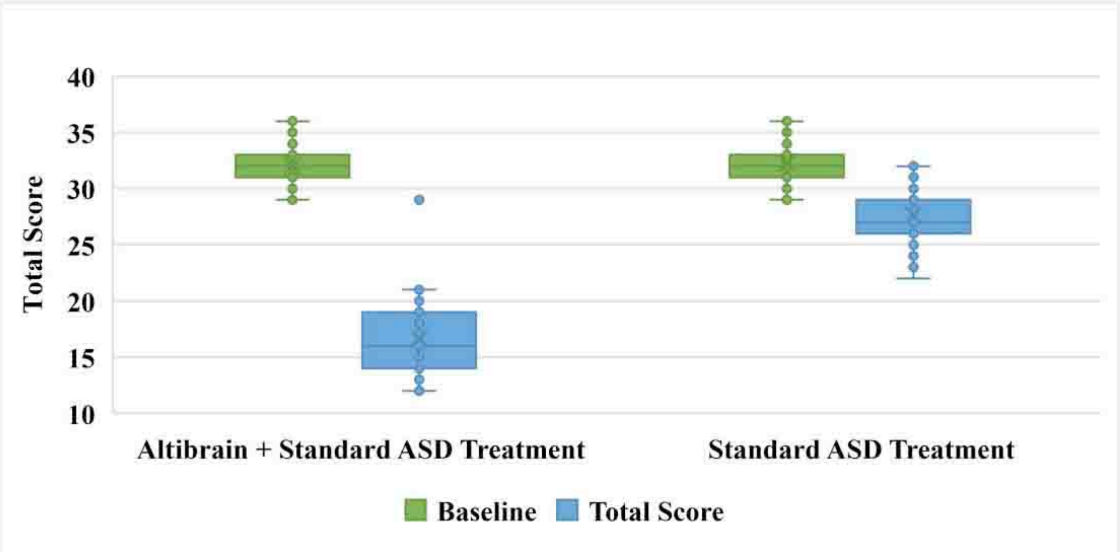

Figure 15: Comparative Analysis of Changes in Part -I Severity of CGI with Altibrain® + Standard ASD Treatment vs. Standard ASD Treatment.

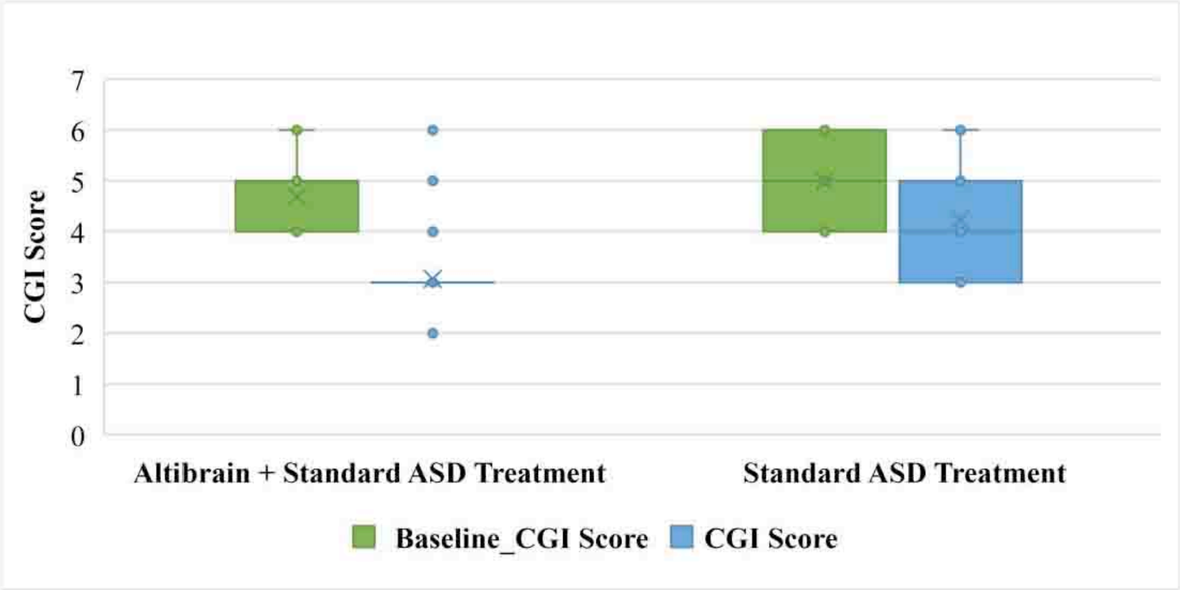

Supplement: Supplementary file 1 — Supplementary material is available on the publisher’s website along with the published article. [file CPD-31-17-1388_SD1.pdf]
